# In and out among the Hospitals

**Published:** 1888-11-17

**Authors:** 


### In and Out Among the Hospitals.

By a Secretary of Ten Years' Standing.

### ROYAL INFIRMARY, DUNDEE.

The work done at the Dundee Hospital is of an interesting nature. There was a daily average of 150 patients, and much valuable information is given in the report as to their treatment. A large number of operations were performed, and the long stay of each patient in the wards is accounted for by the serious nature of the cases treated. The revenue has declined, but fortunately the expenditure has done so too; and as there was but a small sum spent for extraordinary outlays, the managers have been enabled to slightly reduce a debt of £2,500 which burdens the infirmary. There are few provincial hospitals where the details of management are so carefully attended to, and where the efficiency of the establishment is so well maintained. This is in a great measure due to the zeal and energy of the Medical Superintendent, Dr. M'Cosh, and in the directors' report a well-merited recognition is given to the valuable services he has rendered to the hospital. The directors also mention the satisfactory way in which Miss Finlason, the matron, has discharged her duties. One of our correspondents who visited this hospital recently was, however, convinced that economy (?) has been carried too far. There is an air of poverty about the buildings, and especially the wards, which gives a visitor the impression that he is in a workhouse, where no money is spent that can possibly be helped. "Dreary, neglected, and repelling," are the adjectives he uses to describe the wards; and as he has had a unique experience in hospital management, we advise the directors to spent about £8 per bed per annum more, and so bring their institution to a higher level of efficiency and comfort. If they do not do so, they will do much to retard the enforcement of proper economy in other similar charities. There are four medical officers in connexion with the hospital, who visit patients at their own homes. To each of these a district is allotted, and they have altogether attended during the last twelve months 7,478 patients, all of whom were supplied with medicine at the expense of the infirmary. The number of in-patients was 2,052, and out and home patients 11,128; while the expenditure amounted to £7,489 2*s.* 5*d.*, and the receipts to £7,705 18*s.* 7*d.* Allowing 1*s.* for each out-patient, the cost per bed occupied was £42 4*s.* 5*d.* The convalescent home is of great service, and in the year ending June last 989 patients were admitted, two-thirds of whom were sent direct from the infirmary, the others being admitted from among the general public.

### BOLTON INFIRMARY.

Under the able chairmanship of the Rev. Canon Atkinson, M.A., B.C.L., Vicar of Bolton, much good work has been done at the Bolton Infirmary and Dispensary during the year ending March last. A total of 5,456 persons received the benefits of the charity; of these 684 were in-patients, 2,323 were out-patients and casualties, and 597 patients were treated by the hospital staff at their own homes. The hospital now contains 11 beds, and there was a daily average of 720 patients in the wards, of which 26 were children. The total receipt was £7,362 3*s.* 4*d.*, which included legacies amounting to £3,427 16*s.* 7*d.*, and the expenditure £4,487 16*s.* 1*d.* This gives the cost per bed occupied, after deducting 1*s.* for each out and home patient, at £55 5*s.* 10*d.* The new heating apparatus works well, and is a source of much comfort. In this infirmary the chaplain's appointments, like that of any member of the medical staff, are honorary, and the committee, in acknowledging their obligation to the clergy and ministers who have attended, record their sense of the valuable services rendered for the spiritual welfare of the patients.

